# Virus-resistant pigs might help to stem next outbreak

**DOI:** 10.7554/eLife.09790

**Published:** 2015-07-29

**Authors:** Lisbeth Ramirez-Carvajal, Luis L Rodriguez

**Affiliations:** Plum Island Animal Disease Center, Agricultural Research Service, US Department of Agriculture, Orient Point, United States and Oak Ridge Institute for Science and Education, PIADC Research Participation Program, Oak Ridge, Tennessee, United States; Plum Island Animal Disease Center, Agricultural Research Service, US Department of Agriculture, Orient Point, United Statesluis.rodriguez@ars.usda.gov

**Keywords:** FMDV, transgenic pigs, RNAi, viruses

## Abstract

By genetically engineering pigs to degrade a crucial viral protein, livestock can be made less susceptible to foot and mouth disease virus.

**Related research article** Hu S, Qiao J, Fu Q, Chen C, Ni W, Wujiafu S, Ma S, Zhang H, Sheng J, Wang P, Wang D, Huang J, Cao L, Ouyang H. 2015. Transgenic shRNA pigs reduce susceptibility to foot and mouth disease virus infection. *eLife* **4**:e06951. doi: 10.7554/eLife.06951

**Image** Pig cells showing signs of foot and mouth disease infection (top panels); genetic techniques can be used to make cells less susceptible to the infection (bottom panels)

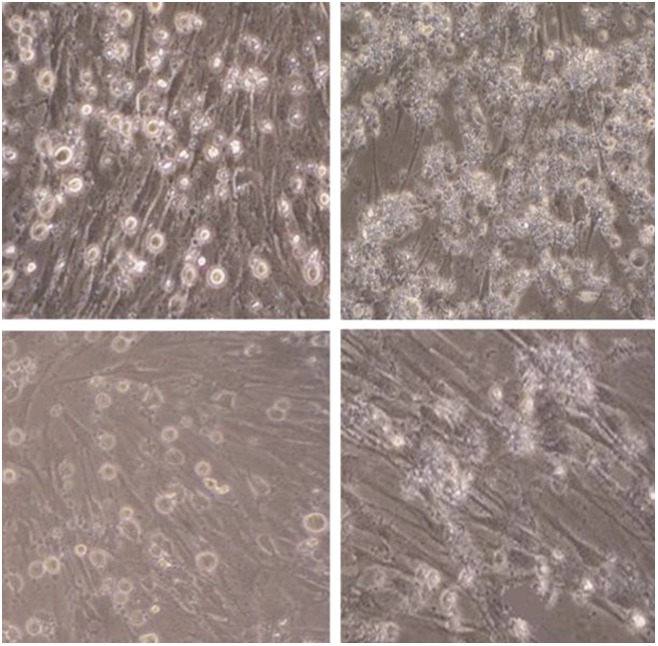

In 2001, an epidemic of foot and mouth disease in the UK resulted in the destruction of over six million livestock, restrictions on international trade, and economic costs in excess of £3 billion (Thompson et al., 2002). Foot and mouth disease is most common in developing areas of the world, such as parts of Asia, Africa and the Middle East, and an outbreak of the disease in animals raised as food resources in such areas can have a devastating effect on economic growth. Now, in eLife, researchers at Shihezi University, Jilin University and the Xinjiang Academy of Animal Science, all in China, report the results of experiments on genetically engineered pigs that could have broad implications for the control of the disease (Hu et al., 2015).

The virus that causes foot and mouth disease (FMD) comes in seven types, each of which requires a specific vaccine for immunization (Grubman and Baxt, 2004). The Chinese team focused on a protein called VP1 that is variable across all different FMD viruses. VP1 is part of the viral capsid, a three-dimensional array of proteins that is involved in receptor binding and entry into host cells (Mason et al., 2003). Hu et al. designed small RNA molecules that silenced the gene for VP1 via a process called RNA interference (Fire et al., 1998). Having less VP1 might decrease the number of viral particles that are produced and make FMD virus less able to spread between animals.

The Chinese team - which includes Shengwei Hu, Jun Qiao and Qiang Fu as joint first authors, and Chuangfu Chen, Wei Ni and Hongsheng Ouyang as corresponding authors - looked for the small RNA that was best able to stop the replication of the FMD virus. They then integrated the gene coding for this small RNA into the genome of single pig cells; transgenic embryos were then generated from these cells by the same technique that produced Dolly the sheep. The embryos were subsequently implanted into donor sows that gave birth to transgenic piglets.

Hu et al. observed that the FMD virus was almost completely inhibited from growing and dividing inside the cells derived from transgenic pigs. The antiviral activity of the small RNA was confirmed when genetically modified pigs were directly injected with one of the FMD virus types common in Asia. Whereas control groups displayed the classical clinical signs of FMD - high fever, mouth blisters, foot sores and lameness - all transgenic pigs were protected from disease for up to seven days after infection, and only later developed milder clinical signs associated with FMD. It remains to be shown if protection extends to other types of FMD virus.

The FMD virus replicates quickly and mutations accumulate when it duplicates its genome. If mutations appear in the region of the genome targeted by the small RNAs, variants that are resistant to treatment can emerge: such ‘escape mutants’ have been observed for other viruses that are similar to the FMD virus (Gitlin et al., 2005). Hu et al. did not examine the viruses recovered from the transgenic pigs, so they did not identify escape mutants. However, if escape mutants do turn out to be a problem, one solution would be to use several small RNAs to target multiple regions of the virus genome at the same time. Since the target region is different among the different types, multiple small RNAs could also be combined to target different types of FMD virus. This would decrease the chances of the viruses gathering exactly the right mutations to evade RNA interference and could extend protection to other types of FMD virus.

Current FMD vaccines require at least seven days to induce a protective immune response (Golde et al., 2005), and during this period animals are susceptible to FMD virus infection. The ability of the virus to rapidly replicate helps it to spread and evade the immune response of its host. Importantly, Hu et al. reported that the amount of virus found in the blood or blisters of infected transgenic pigs was much lower than in the control group, suggesting that the transgenic pigs are less contagious and would be less likely to spread the disease. In the case of an outbreak, genetically engineered pigs would be less susceptible to the infection, would shed fewer virus particles and would slow down the spread of the infection. This would give animal health authorities the necessary time to implement control programs such as vaccination. The downside of this approach is that clinical signs of infection would be masked or undetected: this means that the introduction of transgenic pigs would have to be accompanied by the introduction of robust disease surveillance systems.

The work of Hu et al. is a proof of concept for the use of RNA interference technology to produce transgenic livestock with increased resistance to viral infection. Although this technology could potentially increase food security, it also faces major challenges since, to date, neither the Food and Drug Administration in the United States nor the European Food Safety Authority have approved genetically engineered animals for human consumption. The processes used by these regulators to ensure the safety of genetically modified animals as food resources are strenuous and expensive, and have prevented other transgenic animals, such as salmon, from entering the market in the US (Ledford, 2013). A global consensus on the regulation of genetically engineered organisms has not been reached and each country adopts policy at a national level. However, the governments in a number of countries - including Argentina, Brazil and China - view transgenic products as a way to resolve food security issues. Such countries have simpler protocols for the approval of transgenic products, which might allow transgenic animals to be approved for human consumption (Maksimenko et al., 2013).
